# Mastery learning improves students skills in inserting intravenous access: a pre-post-study

**DOI:** 10.3205/zma001055

**Published:** 2016-08-15

**Authors:** Hendrik Friederichs, Britta Brouwer, Bernhard Marschall, Anne Weissenstein

**Affiliations:** 1 University of Muenster, Studienhospital, Muenster, Germany; 2 University of Muenster, Institute of Medical Education – IfAS, Muenster, Germany

**Keywords:** mastery learning, undergraduate medical education, peripheral venous catheter, medical students

## Abstract

**Objective: **Inserting peripheral venous catheters (PVCs) has been identified as a core competency for medical students. Because the performance – even of hygienic standards – of both students and novice physicians is frequently inadequate, medical faculties must focus on competence-based learning objectives and deliberate practice, features that are combined in mastery learning. Our aim was to determine the competency of students in inserting PVCs before and after an educational intervention.

**Design: **This study comprised a skills assessment with pre- and post-tests of a group of third-year students who received a simulation-based intervention. A newly established curriculum involved one hour of practice at inserting PVCs on simulators. Students were required to pass a test (total 21 points, pass mark 20 points) developed on the concept of mastery learning. An unannounced follow-up test was performed one week (8 days) after the intervention.

**Setting: **The simulation center of the medical faculty in Muenster.

**Participants: **Third-year students who received the intervention.

**Results: **One hundred and nine complete data sets were obtained from 133 students (82.5%). Most students (97.2%) passed the test after the intervention (mean score increase from 15.56 to 20.50, *P*<0.001). There was a significant decrease in students’ performance after one week (8 days): only 74.5% of participants passed this retest (mean score reduction from 20.50 to 20.06, *P*<0.001).

**Conclusion: **Mastery learning is an effective form of teaching practical skills to medical students, allowing a thorough preparation for the challenges of daily clinical practice.

## Zusammenfassung

**Hintergrund: **Das Legen von peripheren Venenverweilkanülen ist als Kernkompetenz von Medizinstudierenden bestimmt worden. Da die diesbezügliche Performance – auch im Hinblick auf hygienische Standards – häufig inadäquat ist, müssen sich medizinische Fakultäten mehr auf kompetenz-basierte Lernziele und “deliberate practice” fokussieren. Diese Aspekte sin im Mastery Learning kombiniert. Unser Ziel war es, die Kompetenz im Legen von peripheren Venenverweilkanülen nach einer Lehrintervention zu messen.

**Studiendesign:** Diese Studie umfasst eine Vorher-Nachher- Kompetenzmessung von Studierenden im 3. Studienjahr, die eine simulations-basierte Lehrintervention erhielten. Die Studenten mussten einem Test bestehen (insgesamt 21 Punkte, Mindestpunktzahl 20 Punkte), welcher basierend auf dem Konzept der Mastery Learning entwickelt wurde. Ein unangekündigter Follow-up- Test wurde eine Woche (8 Tage) nach dem Eingriff durchgeführt.

**Rahmen:** Das Studienhospital der Medizinischen Fakultät Münster.

**Teilnehmer: **Teilnehmer waren Studierende im 3. Studienjahr.

**Ergebnisse: **Es konnten 109 vollständige Datensätze von 133 Studierenden (82,5%) in die Analyse einbezogen werden. Die meisten Studierenden (97,2 %) haben den Test nach der Lehrintervention bestanden (durchschnittliche Erhöhung der Gesamtpunktzahl von 15,56 auf 20,50, *P*<0,001). Es gab eine signifikante Abnahme der Performance der Studierenden nach einer Woche (8 Tage): nur 74,5% der Teilnehmer bestanden diesen erneuten Test (mittlere Reduktion der Gesamtpunktzahl von 20,50 auf 20,06, *P*<0,001).

**Schlussfolgerung: **Mastery Learning ist ein effektives Mittel um Medizinstudierenden praktische Fertigkeiten beizubringen und ermöglicht damit eine umfassende Vorbereitung auf die Herausforderungen des klinischen Alltags.

## Introduction

At present, the requirements of physicians are that they are scientifically and practically trained, able to practice medicine, and undertake training and continuing education independently and responsibly (http://www.gesetze-im-internet.de/_appro_2002/BJNR240500002.html cited 4. Dezember 2012). Accordingly, the goal of undergraduate medical education is to enable future physicians to act in a competent and responsible manner. Therefore, medical education should be performed on a scientific basis and be related to medical practice as well as to patients. Despite these requirements, considerable deficits in the competence and performance of students as well as novice physicians are often noted [1], [2]. In addition, a repeated criticism is that education by medical schools is too theoretical, emphasis being primarily on the transmission of knowledge-based content [3]. In response to this criticism, a shift from purely cognitive to more competence-based learning objectives in medical education is occurring internationally [4]. 

A methodology in providing competence-based education in medicine is mastery learning. First described by Bloom in 1968, this concept means that learners acquire knowledge and skills, which are measured against fixed achieved standards, the time taken to reach the required outcomes being irrelevant [5], [6]. The aim of mastery learning is to ensure that all learners reach teaching objectives with little or no outcome variance [7]. It describes a process of mastering specific learning objectives and requires clearly defined teaching aims organized into small, successively organized units; students are not only required to understand the material before moving to the next lesson [8], but to achieve a defined proficiency before proceding [9]. Various studies in postgraduate medical education have examined the acquisition of skills involved in performing invasive procedures and proven the effectiveness of this method. Among others, there is such evidence for learning how to perform extended resuscitation [10], central venous catheter insertion [11], lumbar puncture in infants [12] and laparoscopic techniques [13]. So far, only one study has described implementation of mastery learning in undergraduate medical education [14]. To our knowledge, the mastery learning method has never been used to teach an invasive procedure to undergraduate medical students. We have therefore conducted a study to integrate and evaluate this type of educational intervention in the medical curriculum.

In this study, we implemented the concept of mastery learning in the course “Medical Skills Lab”, which took place in the simulation center of the medical faculty (Studienhospital Muenster^®^). The framework was insertion of peripheral venous catheters (PVCs), a core clinical skill that students need to acquire. Given that 5-7% of all PVCs have reportedly become colonized by bacteria by the time catheter material [15], [16] and PVC-associated septicemia occurs in up to 2% of all patients with PVCs [16], [17], it is not surprising that insertion and care by specially trained personnel is associated with a significant reduction in occurrence of phlebitis and infection [18], [19]. Thus, in regard to patient safety, it is desirable that medical students are able to perform this task as well as possible. The aims of our study were as follows:

**Primary Outcomes**

1. To assess whether mastery learning increases third-year (fifth semester) students’ skills in inserting PVCs

**Secondary Outcomes**

1. To evaluate third-year students’ skills in inserting PVCs one week (8 days) after the intervention
2. To evaluate the feasibility of integrating mastery learning permanently into the medical curriculum

## Methods

### Study Design

A skills assessment with pre- and post-tests of a group of third-year students was performed using a simulation-based intervention. Participants’ skills in placing PVCs were assessed before and immediately after the course (on the same day) in the form of pre- and post-tests, which were part of the intervention. Follow-up tests were performed eight days after the intervention. Further, relevant data (age, number of clinical internships, and number of previously placed PVCs) were collected from participants.

#### Participants 

From December 2012 to January 2013, the proficiency of 133 third year students at inserting PVCs was assessed before and after the intervention. The students had not received any formal training. In Germany, medical students are required to complete six years of study (12 semesters); thus, the students had already completed their preclinical stage (first two years) and entered the clinical phase of their studies. All participants gave their informed consent before the pre-test. The Ethics Committee of the Chamber of Physicians, Westfalen-Lippe, and the Medical Faculty of the Westfalian Wilhelms University, Muenster, waived requirements for an ethical approval procedure.

#### Outcome Measures and Measuring Instrument

The primary outcome measure was comparison of the students’ test scores. All three tests (pre-test, post-test, follow-up test) had the same structure and were part of the intervention. They further served as an assessment tool in the form of a checklist, thus providing a guide for the correct insertion of PVCs. The checklist was divided into four units: preparing materials, preparation of the puncture, execution of the puncture, and finally fixation of the venous catheter. Each unit was further subcategorized into smaller, successively organized subunits (seven, four, five and five subunits, respectively). For example, subunits included retrieving gloves, disinfection of the puncture site and announcing the puncture to the patient. The structure and sequence of all units were developed in accordance with the objective structured clinical examination checklist of Juenger and Nikendei [20]. Each successfully performed sub-unit scored one point. Thus, 21 points could be scored; students passed the test once they had scored 20 points. For reasons of patient safety the pass mark in this study was defined as 20 points; thus, it was not possible to pass without correctly disinfecting the skin. After the course, the absolute passing score for the performance examination was defined by a multidisciplinary panel of eight clinical experts, who determined the minimum passing score by the Hofstee (group-based) standard setting method [21]. The panel consisted of faculty members who were certified in family medicine (*n*=2), surgery (*n*=1), orthopedics (*n*=1), internal medicine (*n*=1) and three faculty members involved in medical education. Each panelist received instructions in a standard setting. As a result, a Hofstee score of 20 points was used as the final minimum passing score.

#### Educational Intervention 

The newly developed course consisted of one-hour practice at inserting PVCs on simulators (IV-arm, Carl Gustav Carus Management GmbH, Dresden, Germany). Students were asked to imagine caring for a real patient and to provide the imaginary patient while commenting each step of their actions. A pre-test was performed for baseline assessment. After the pre-test, the students practiced inserting PVCs in groups of two on simulators for a total of 1 hour (training period of 30 minutes per student) and the deficits noted in the pre-test were discussed. The training sessions were supported by peer-teachers (trained student tutors from higher semesters). Each tutor had received thorough instructions for about one hour and schooling on the checklist prior to the study. 

A post-test was performed at the end of the educational intervention. Eight days after the course, an unannounced voluntary follow-up test was offered, allowing students to assess their skills again. 

#### Data Processing and Analysis

Only students with complete data sets (i.e., who had participated in both pre- and post-tests) were included in the analysis. Participants who passed the pre-test on their first attempt were excluded from the study because the desired outcome measure was an increase in students’ skills after the intervention, and participants who had passed the pre-test could not further improve their performance. Our primary analysis was a comparison of the mean total test scores (pre-test, post-test) and comparison of follow-up test scores. Data were analyzed with IBM Statistics SPSS 19. Descriptive means and standard deviations were calculated and other parametric tests used. Student’s paired t-test was performed to compare the results of pre- and post-tests and of post- and follow-up tests. Pearson's χ^2^ test was used to compare pass rates. Further, one-way ANOVA was performed in order to evaluate differences between the groups (significance level *P*≤0.05). We used the standard alpha-level of 0.05 for significance and a power level of 0.8. Thus, we needed a sample size of at least 27 participants to detect a medium effect size (d=0.5) (G*Power 3.1).

## Results

### Participants

In all, 133 third-year students performed the pre-test. After exclusion of 16 students who passed the pre-test on their first attempt and a further 8 whose records were incomplete, the final sample comprised 109 third-year students. Baseline characteristics of partcipants are described in Table 1 (Tab. 1). Of the 109 third-year students enrolled in the study, 106 completed the follow-up test.

#### Primary Outcomes

Following the educational intervention, 106/109 students (97.2%) passed the test with a mean score (standard deviation) of 20.50 (0.56), whereas the mean score at baseline was 15.56 (2.44). This improvement in the students’ performances is highly significant (P<0.001; effect size Cohens' d 2.79). The remaining three students scored 19 points and had the opportunity to repeat the course until they passed the test after the study.

#### Secondary Outcomes

**Figure 1:**
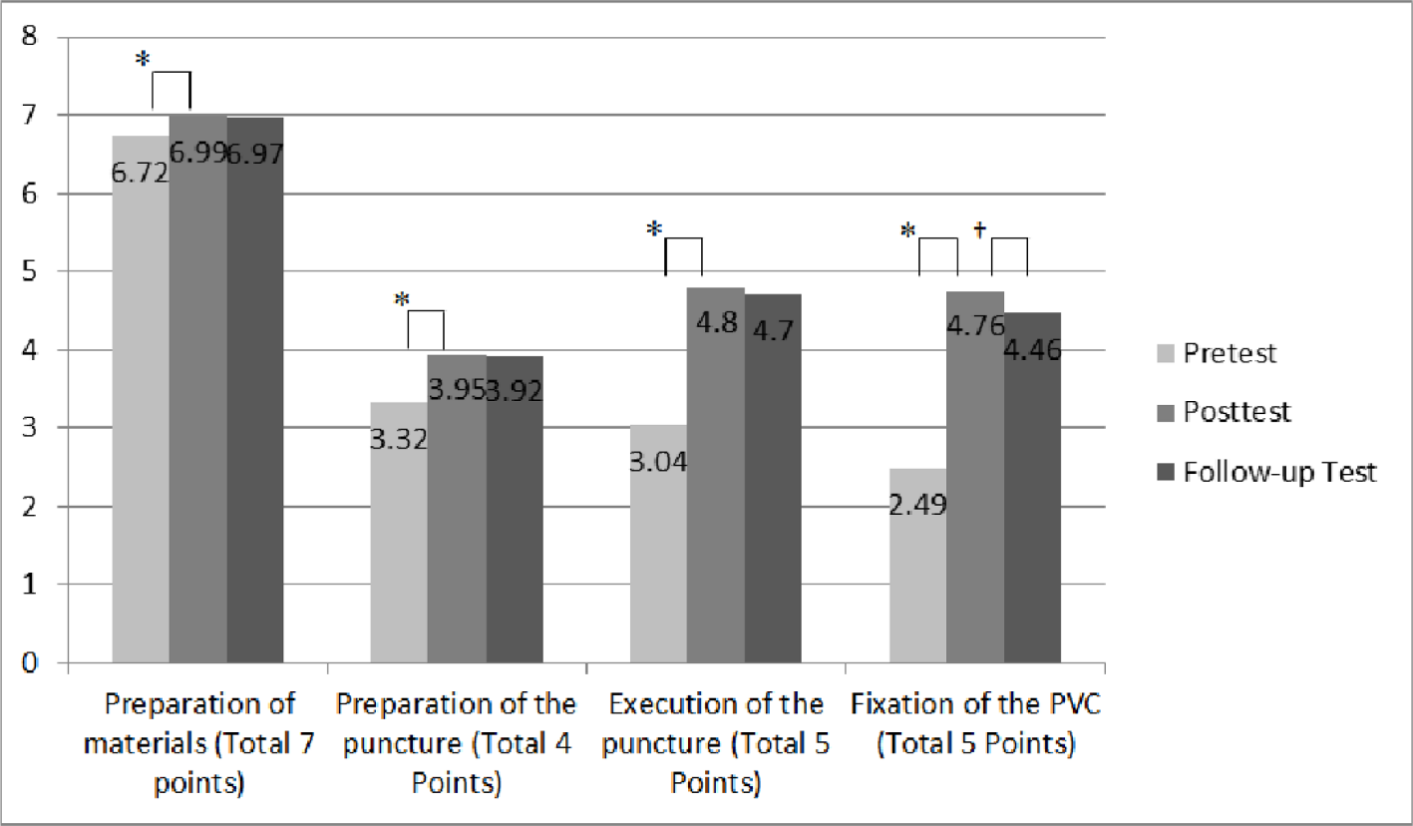
Performance of students according to the sub-units of the task in the pre- and post-tests (n=109) and one week (8 days) after the educational intervention (follow-up test, n=106). *Significant differences between the pre- and post-test results for all four subunits (P<0.05). †Significant difference between post- and follow-up test for “fixation of the PVC” only (P<0.05).

About one week (8 days) after the educational intervention, 106 students performed a follow-up test, in which they scored significantly less (20.06 [0.94] points) than in the previous test (post-test) (20.50 [0.56] points) (P < 0.001). The effect size (Cohen's d) in comparison to the mean score at baseline is 2.43. However, with the pass mark being 20 of a possible 21, three-quarters of the students (74.5%) still passed the test (20.8% of students scored 19 points, 2.8% scored 18 and 1.9% scored 17). The performance of students according to sub-units of the task is depicted in Figure 1 (Fig. 1). 

## Discussion

Our results show that our educational intervention significantly increased students’ practical skills in inserting PVCs. After the course, the majority of students (97.2%) passed the test. However, there was a significant decrease in students’ performances after one week (8 days), only 74.5% of participants passing the test after this interval (mean score reduction from 20.50 to 20.06 points). 

Inserting PVCs is a core clinical skill students need to possess and physicians expect them to perform this task during their clinical internships. Most patients are willing to let medical students perform even invasive procedures after simulator-training [22]. thus medical schools have the responsibility of preparing students in the best possible way. In accordance with the concept of mastery learning, our intervention focused on clearly defined goals, deliberate practice and precise outcome measures. Another advantage of this method is that, because the outcome measures are standardized, students’ learning is no longer dependent on the teaching skills of tutors. Further, self-reflection is a key aspect of mastery learning, thus value was set on the active articulation of performed steps. Mastery learning allows students to learn and practice invasive diagnostic and therapeutic procedures with unprecedented frequency. This concept has become an important component of continuing education because it has been shown to improve the quality of patient care [11], [13], [23], [24]. In our opinion the key element of mastery learning of clinical skills is that learners receive individual, immediate feedback according to transparent standards, allowing them to focus on their own learning needs. It seems logical that this concept be applied to medical education for the benefit of students and, of course, the patients treated by them. 

The results of our study should be compared with the results from a review of mastery simulation-based medical education (SBME) of Cook who states that “In comparison with no intervention, mastery SBME what associated with large effects on skills (41 studies; effect size [ES] 1:29 [95% confidence interval, 1:08 to 1:50])” [9]. Our effect size showed with a value of 2.79 in the post-test and 2.43, eight days later even higher results. Due to the quality of data and analysis the evidence of a meta-analysis is of course significantly higher than the results of our study. But in a subgroup analysis of the described review only 6 trials with a total of 109 medical students were eligible. For them, the effect size was 1.2 (95% CI 0.44 to 1.95) [Attachment Digital Table 2 in this publication ]. We could already include 106 participants in our study.

Our study has several limitations. First, the pre- and post-tests were performed on the same day and, because of the course structure, it was not possible to blind the tutors according to pre-, post- and follow-up tests. Second, three third-year students did not pass the post-test; however, the concept of mastery learning dictates that all learners reach the learning objectives. Therefore, after the study these students had the opportunity to repeat the course until they succeeded.

How D.A. Cook in his work “If you teach them, they will learn” [25] discussed, studies describing the impact of a single specific training have only a small impact for educational research and the actual teaching. Accordingly, in future a controlled study, ideally even a RCT should be conducted to determine the effectiveness of the teaching intervention in more detail.

Despite these limitations, our study has shown that mastery learning is an effective form of teaching practical skills during the education of medical students. We hope to further demonstrate that this teaching format could be introduced to continuing graduate education as well as undergraduate medical education. In light of the promising results of our study, both regarding the efficiency of the teaching units and the favorable feedback from students, we have integrated the concept of mastery learning into the medical curriculum on a long-term basis. Mastery learning is a valuable model that helps to prepare medical students for the challenges of daily clinical practice and thus facilitate their entry into professional life.

## Authors’ contributions

HF conceived of the study and participated in data acquisition, data analysis and the drafting of the manuscript. BB participated in data acquisition and data analysis. BM participated in coordination. AW participated in data analysis and helped to draft the manuscript. All authors have read and approved the final manuscript.

## Author information

HF is the head of the study hospital of the medical school in Muenster and has a master’s degree in Medical Education (MME). BM is the Dean of the medical faculty and head of the Institute of Medical Education and Student Affairs. AW and BB have medical degrees and work as research assistants in the study hospital.

## Competing interests

The authors declare that they have no competing interests.

## Supplementary Material

Digital Table 2

## Figures and Tables

**Table 1:**
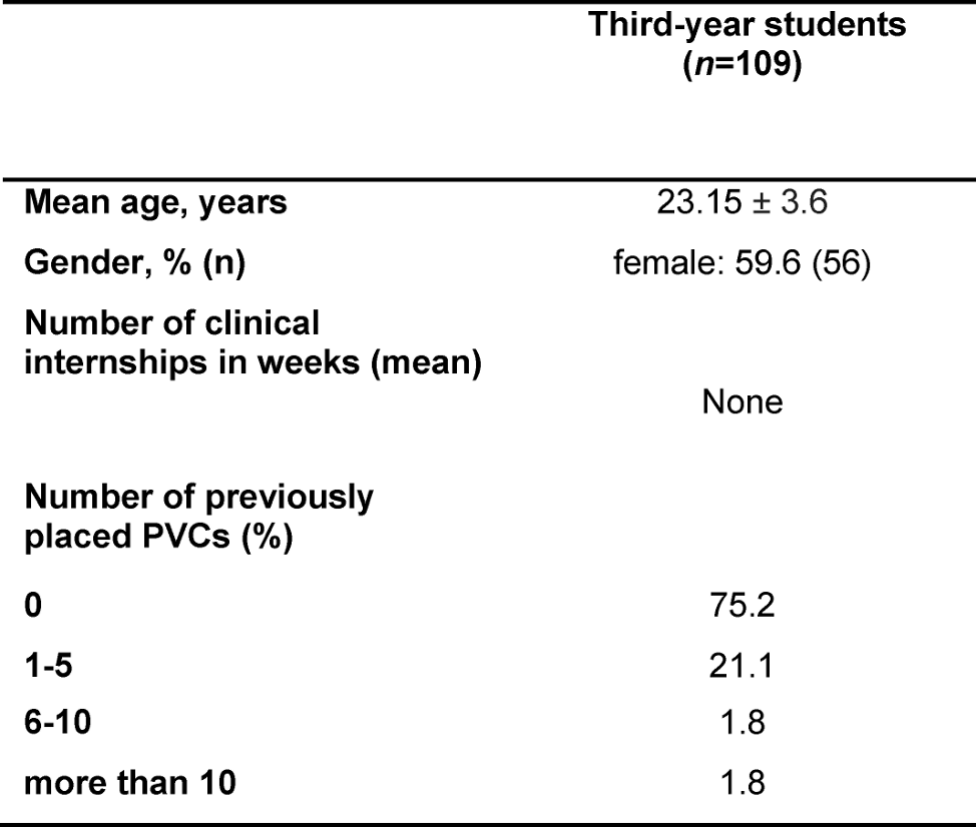
Participants’ characteristics at baseline
