# Supplementary material for: Mastery learning improves students skills in inserting intravenous access: a pre-post-study
Source: GMS J Med Educ. 2016 Aug 15;33(4):Doc56. doi: 10.3205/zma001055 (PMC5003138; doi:10.3205/zma001055)
Supplement: Digital Table 2 [file JME-33-56-s-001.pdf]

Supplemental Digital Table 2

Subgroup Analyses of Studies Comparing Simulation-Based Mastery Learning with No Intervention *

Table 2.1. Subgroup Analyses for Outcome: Time Skill

Feature	Subgroup	No. studies (No. participants)	ES	95% confidence interval	$P_{interaction}$
All		15 (278)	0.94	0.64, 1.24	
No. groups, timing	2-group posttest-only	3 (52)	1.18	0.58, 1.79	
	2-group pre-post	6 (151)	0.76	0.3, 1.22	
	1-group pre-post	6 (75)	1.09	0.64, 1.54	
Group allocation	Randomized	8 (171)	0.68	0.36, 1.01	0.004
	Nonrandomized	1 (32)	1.62	1.07, 2.17	
Participants	Medical student	6 (109)	1.2	0.44, 1.95	
	Postgraduate trainees	11 (212)	1.03	0.71, 1.35	
	Practicing physicians	1 (18)	1.37	0.65, 2.08	
	Other	1 (14)	1.79	0.45, 3.12	
Cognitive interactivity	High	13 (258)	0.9	0.6, 1.2	0.52
	Low	2 (20)	1.59	-0.51, 3.69	
Feedback	High	2 (25)	0.98	0.43, 1.53	0.9
	Low	13 (253)	0.94	0.61, 1.27	
Repetitions	Many >10	12 (225)	0.81	0.51, 1.1	0.01
	Few ≤9	3 (53)	1.56	1.09, 2.03	
Time learning	≥5 hours	5 (129)	1.11	0.56, 1.67	0.64
	<5 hours	9 (130)	0.96	0.65, 1.27	
Assessment	Blinded	12 (221)	1.05	0.73, 1.37	0.28
	Not blinded	3 (57)	0.61	-0.13, 1.34	
NOS	High ≥4	6 (144)	0.56	0.26, 0.86	0.003
	Low <4	9 (134)	1.25	0.89, 1.6	
MERSQI	High ≥12	12 (240)	1.03	0.65, 1.4	0.36
	Low <12	3 (38)	0.77	0.36, 1.18	
Effect size estimation	Precise	13 (245)	0.94	0.6, 1.28	
